# Preferential subcortical collateral projections of pedunculopontine nucleus-targeting cortical pyramidal neurons revealed by brain-wide single fiber tracing

**DOI:** 10.1186/s13041-022-00975-y

**Published:** 2022-10-29

**Authors:** Qiao-Qiong Liu, Yu-Xiao Cheng, Qi Jing, Ke-Ming Zhang, Lu-Feng Ding, Xiao-Wei Fan, Chun-Hui Jia, Fang Xu, Guo-Qiang Bi, Pak-Ming Lau

**Affiliations:** 1 Division of Life Sciences and Medicine, University of Science and Technology of China, Hefei, 230026 Anhui China; 2 Interdisciplinary Center for Brain Information, The Brain Cognition and Brain Disease Institute, Shenzhen Institute of Advanced Technology, Chinese Academy of Sciences, Shenzhen, 518055 Guangdong China

**Keywords:** Pedunculopontine nucleus, CaMKIIα-positive neuron, Collateral projection, Anterior cingulate area, Prelimbic region, Motor cortex, VISoR whole-brain imaging

## Abstract

The pedunculopontine nucleus (PPN) is a heterogeneous midbrain structure involved in various brain functions, such as motor control, learning, reward, and sleep. Previous studies using conventional tracers have shown that the PPN receives extensive afferent inputs from various cortical areas. To examine how these cortical axons make collateral projections to other subcortical areas, we used a dual-viral injection strategy to sparsely label PPN-targeting cortical pyramidal neurons in CaMKIIα-Cre transgenic mice. Using a high-speed volumetric imaging with on-the-fly-scan and Readout (VISoR) technique, we visualized brain-wide axonal projections of individual PPN-targeting neurons from several cortical areas, including the prelimbic region (PL), anterior cingulate area (ACA) and secondary motor cortex (MOs). We found that each PPN-projecting neuron had a unique profile of collateralization, with some subcortical areas being preferential targets. In particular, PPN-projecting neurons from all three traced cortical areas exhibited common preferential collateralization to several nuclei, with most neurons targeting the striatum (STR), lateral hypothalamic area (LHA) and periaqueductal gray (PAG), and a substantial portion of neurons also targeting the zona incerta (ZI), median raphe nucleus (MRN) and substantia nigra pars reticulata (SNr). Meanwhile, very specific collateralization patterns were found for other nuclei, including the intermediate reticular nucleus (IRN), parvicellular reticular nucleus (PARN) and gigantocellular reticular nucleus (GRN), which receive collateral inputs almost exclusively from the MOs. These observations provide potential anatomical mechanisms for cortical neurons to coordinate the PPN with other subcortical areas in performing different physiological functions.

**Supplementary Information:**

The online version contains supplementary material available at 10.1186/s13041-022-00975-y.

The pedunculopontine nucleus (PPN), a heterogeneous structure located in the dorsal tegmentum of the brainstem, is highly conserved across animal species and is known to be involved in several brain processes, including locomotion, reward, motivation, arousal, and behavioral state control [1]. Consistent with its diverse functionality, the PPN is connected to many cortical and subcortical areas; alterations in these connections could underlie brain disorders such as Parkinson’s disease [1, 2]. Anatomical studies have established that the PPN receives direct inputs from the motor cortex and several nonmotor cortical areas, including areas in the prefrontal cortex and the cingulate cortex [3, 4], which are known for their critical roles in reward and motivation [5, 6]. Meanwhile, cortical neurons are known to branch out and project to multiple targets [7]. Thus, it is possible that PPN-targeting axons send collaterals to other subcortical areas, especially those with related functions. In this study, we performed dual-AAV injections to sparsely label PPN-projecting cortical pyramidal neurons in several cortical areas of CaMKIIα-Cre mice. We then used a high-speed volumetric imaging with on-the-fly-scan and Readout (VISoR) technique to obtain the brain-wide 3D morphology of individual neurons targeting the PPN and to characterize their collateral projections.

We first labeled PPN-projecting excitatory neurons in bulk by injecting rAAV2-retro-EF1α-DIO-EGFP into the PPN of CaMKIIα-Cre mice (Additional File 1: Materials and Methods, Fig. S1). Three weeks after injection, the distribution of EGFP fluorescence was examined across the whole brain using our high-throughput VISoR system, with EGFP-positive neurons observed in many cortical and subcortical regions, indicating that the PPN received inputs from these regions (Fig. 1a; Additional file 1: Fig. S2; Additional file 2: Video S1). In addition to the secondary motor cortex (MOs), which is responsible for planning voluntary movement, the most prominent cortical areas with labeled PPN-projecting neurons were the prelimbic region (PL) of the medial prefrontal cortex and the anterior cingulate area (ACA), which are known for their roles in various functions, including attention, emotion, reward, and learning (Fig. 1b; Additional file 1: Fig. S2). Consistent with previous reports [2–4], EGFP expression was found in many midbrain and hindbrain areas, such as the periaqueductal gray (PAG) and parvicellular reticular nucleus (PARN) (Fig. 1c, d).

**Fig. 1:**
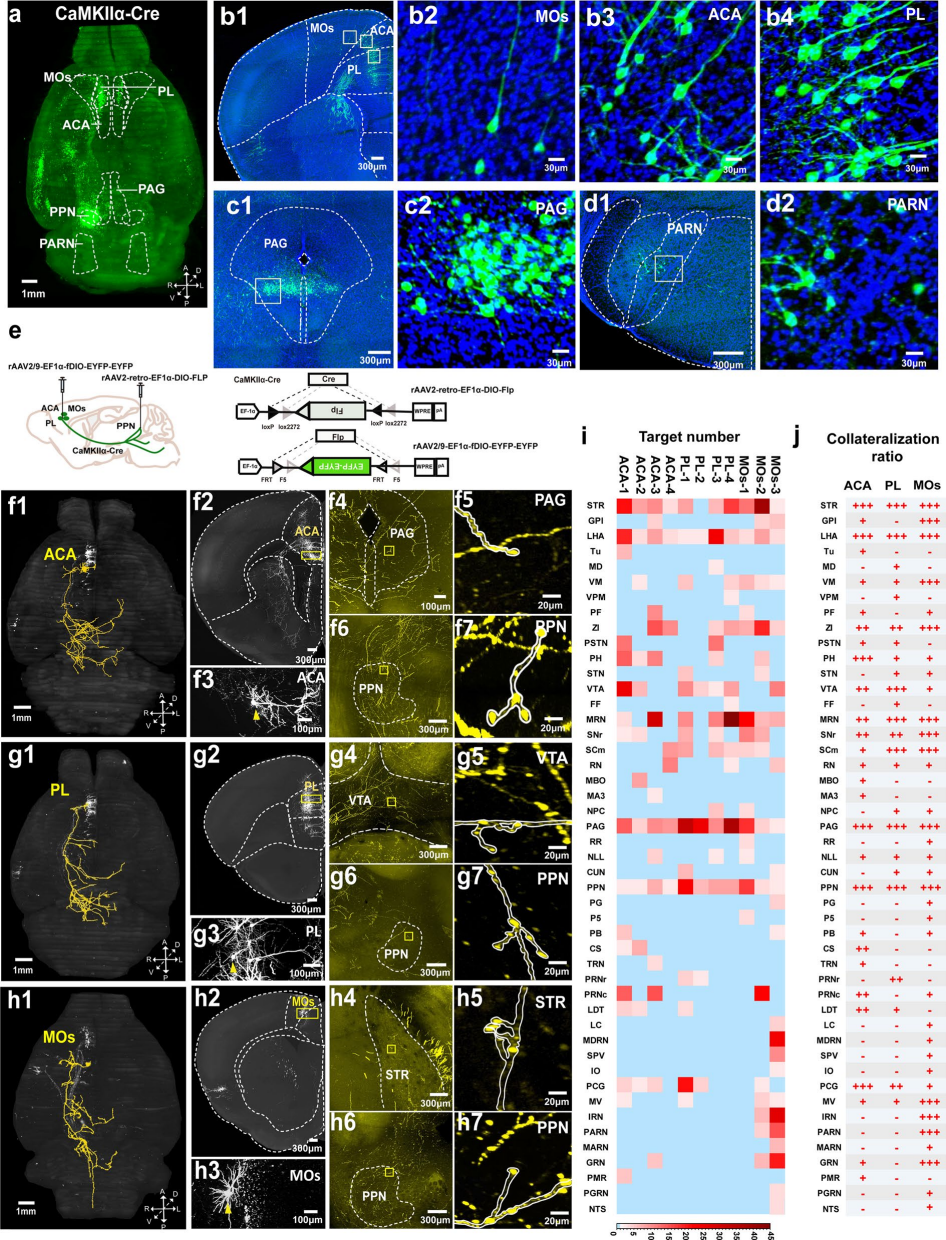
Brain-wide inputs to the PPN and collateralization of PPN-targeting cortical pyramidal neurons. **a** Horizontal view from whole-brain VISoR imaging of excitatory neurons labeled by retrograde tracing virus (rAAV2-retro-EF1α-DIO-EGFP) injected into the PPN of a CaMKIIα-Cre transgenic mouse. Solid lines indicate cortical and subcortical areas to be examined in b and c-d, respectively. A: anterior; P: posterior; L: left; R: right; D: dorsal; V: ventral. **b** Maximum‑intensity projection of part of a 100 μm-thick coronal section encompassing the MOs, ACA, and PL (**b1**). Enlarged views of the boxed areas in b1 show EGFP-expressing PPN-targeting neurons in the MOs (**b2**), ACA (**b3**) and PL (**b4**). **c**, **d** Maximum-intensity projection of part of a coronal section encompassing the PAG (**c1**) and the PARN (**d1**). Enlarged views of the boxed areas in (**c2**) and (**d2**), showing labeled neurons in the PAG and PARN, respectively. **e** Diagrams of the cell type specific and target-specific sparse labeling of PPN-projecting cortical pyramidal neurons. **f–h** Imaging and 3D reconstruction of individual PPN-projecting neurons from the ACA (**f**), PL (**g**), and MOs (**h**), showing brain-wide axonal projections (**f1**, **g1**, **h1**) and dendritic morphologies (**f2**, **f3**, **g2**, **g3**, **h2**, **h3**) of sparsely labeled cortical neurons. Enlarged views of subcortical areas within the PAG (**f4**, **f5**), VTA (**g4**, **g5**), STR (**h4**, **h5**), and PPN (**f6**, **f7**, **g6**, **g7**, **h6**, **h7**) show termini of axonal collaterals from the traced cortical neurons. **i** Collateralization profile of individual pyramidal neurons in the ACA (n = 4), PL (n = 4) and MOs (n = 3). The heatmap shows the number of axon terminals made by each neuron. **j** Qualitative summary of subcortical collateralization from the three cortical areas. The collateralization ratio (CR, defined as the number of PPN-projecting neurons from a given cortical area making at least 2 terminals in a specified subcortical target area divided by the total number of neurons traced for this cortical area) is separated into 3 categories: “−” for CR = 0; “+” for CR ≤ 33.3%; “++” for 33.3% < CR < 66.7%; “+++” for CR ≥ 66.7%

To evaluate how PPN-projecting cortical pyramidal neurons in the ACA, PL and MOs collateralize to other subcortical areas, we employed a sparse labeling strategy to facilitate tracing axonal projections of individual PPN-targeting pyramidal neurons from these three cortical areas. To this end, a low concentration of rAAV2-retro-EF1α-DIO-Flp virus was injected into the PPN of CaMKIIα-Cre mice, with high concentration of rAAV2/9-EF1α-fDIO-EYFP-EYFP virus injected into the PL, ACA, and MOs respectively (Fig. 1e; Additional file 1: Materials and Methods). Four weeks after injection, bright EYFP fluorescence was observed in a small set of neurons in the corresponding cortical areas with 3D VISoR imaging at 1 μm resolution. The sparseness of the labeling allowed us to trace the full morphology of individual axons, including all branches and terminal arborizations for some of these cortical pyramidal cells, which, in addition to the PPN, also made clear collateral projections to other subcortical areas, including the periaqueductal gray (PAG), ventral tegmental area (VTA) and striatum (STR) (Fig. 1f–h; Additional file 3: Video S2, Additional file 4: Video S3, Additional file 5: Video S4, Additional file 6: Video S5, Additional file 7: Video S6, Additional file 8: Video S7). In total, 11 PPN-targeting pyramidal neurons from three imaged animals were traced and verified, with 4 neurons from the ACA, 4 from PL and 3 from MOs (Fig. 1i). Each of these traced neurons formed at least 2 axonal termini in the PPN. For quantification purposes, we defined a subcortical area to be a collateral target of a cortical neuron if two or more axonal termini were formed in this area.

The 11 traced neurons exhibited diverse collateralization profiles, with each individual neuron targeting 6 to 24 subcortical areas (Fig. 1i). Overall, they made collateral projections to nearly 50 subcortical nuclei, some of which emerged as preferential collateralization targets (Fig. 1j). Three subcortical nuclei, including the PAG, STR, and lateral hypothalamic area (LHA), received extensive collateral inputs from a supermajority of PPN-projecting neurons (67% or more, marked “+++” in Fig. 1j) in all three traced cortical areas, with at least two termini formed by each input neuron. Among them, the LHA and PAG both received collateral inputs from all 11 neurons. The STR, LHA, and PAG are known to be important for diverse functions, such as movement control, arousal, reward, and learning [8–11], which the PPN also participates in. Some other subcortical regions received extensive collateral inputs from one or two cortical areas, together with collateral inputs from a smaller but still substantial fraction of neurons (34%–66%, marked “++” in Fig. 1j) in the other cortical areas. These include the zona incerta (ZI), which is involved in eating and defensive behavior; the reticular part of substantia nigra (SNr), which is a major output nucleus of the basal ganglia; and the midbrain reticular nucleus (MRN), which is involved in motor control. Several cortical neurons, such as MOs-2 and PL-3, sent collaterals to all 6 aforementioned nuclei (Fig. 1i). The broad collateral inputs into these nuclei from different cortical areas could play a role in coordinating their related functional activity to support complex behavior.

On the other end of the spectrum, some subcortical regions received collateral inputs primarily from one of the three cortical areas. For example, the intermediate reticular nucleus (IRN), parvicellular reticular nucleus (PARN), and gigantocellular reticular nucleus (GRN) all received extensive collateral inputs from the MOs. However, only a small fraction (no more than 33%, marked “+” in Fig. 1j) or none (marked “–” in Fig. 1j) of the neurons in the ACA and PL made collateral inputs to these three nuclei. The specificity of the collateralization pattern here is in accordance with the rather particular role of these nuclei in motor-related functions [12–14].

Our observations regarding the collateralization patterns were primarily based on a limited number of traced neurons. To further validate these results, we explored the recently published mouse brain projectome database (https://mouse.braindatacenter.cn) [15], from which we searched for PPN-projecting neurons with soma in the three cortical areas above that collateralize to the aforementioned subcortical targets. Even though the projectome data are not strictly pyramidal specific, analysis of the search results largely confirmed the commonality and specificity of collateralization as described above (Additional file 1: Table S1). Through these distinctly preferential collateral projections, different cortical neurons may work synergistically to coordinate the activity of different subcortical neuronal ensembles to control complex cognitive functions and behaviors.

## Supplementary Information

**Additional file 1: Materials and Methods, Supplementary figures S1, S2 and Table S1. Fig. S1. **Microinjection of rAAV2-retro to the PPN of a CaMKIIα-Cre mouse. **Fig. S2. **Brain-wide distribution of PPN-projecting neurons. **Table S1. **Verification of preferred subcortical collateralization of PPN-projecting cortical areas based on mouse brain projectome data.

**Additional file 2: Video S1.** Whole-brain distribution of CaMKIIα afferent neurons to the PPN.

**Additional file 3: Video S2.** Axonal arborizations of ACA neurons in the PPN.

**Additional file 4: Video S3.** Axonal arborizations of ACA neurons in the PAG.

**Additional file 5: Video S4.** Axonal arborizations of MOs neurons in the PPN.

**Additional file 6: Video S5.** Axonal arborizations of MOs neurons in the striatum.

**Additional file 7: Video S6.** Axonal arborizations of PL neurons in the VTA.

**Additional file 8: Video S7.** Axonal arborizations of PL neurons in the PPN.

## Data Availability

All data presented are available upon reasonable request.

## References

[1] Benarroch EE (2013). Pedunculopontine nucleus: functional organization and clinical implications. Neurology.

[2] Pahapill PA, Lozano AM (2000). The pedunculopontine nucleus and Parkinson’s disease. Brain J Neurol.

[3] Monakow KH, Akert K, Künzle H (1979). Projections of precentral and premotor cortex to the red nucleus and other midbrain areas in *Macaca fascicularis*. Exp Brain Res.

[4] Özkan M, Köse B, Algın O, Oğuz S, Erden ME, Çavdar S (2022). Non-motor connections of the pedunculopontine nucleus of the rat and human brain. Neurosci Lett.

[5] Volkow ND, Michaelides M, Baler R (2019). The neuroscience of drug reward and addiction. Physiol Rev.

[6] Anastasiades PG, Carter AG (2021). Circuit organization of the rodent medial prefrontal cortex. Trends Neurosci.

[7] Muñoz-Castañeda R, Zingg B, Matho KS, Chen X, Wang Q, Foster NN (2021). Cellular anatomy of the mouse primary motor cortex. Nature.

[8] Grillner S, Hellgren J, Ménard A, Saitoh K, Wikström MA (2005). Mechanisms for selection of basic motor programs–roles for the striatum and pallidum. Trends Neurosci.

[9] Cox J, Witten IB (2019). Striatal circuits for reward learning and decision-making. Nat Rev Neurosci.

[10] Yu H, Xiang X, Chen Z, Wang X, Dai J, Wang X (2021). Periaqueductal gray neurons encode the sequential motor program in hunting behavior of mice. Nat Commun.

[11] Vázquez-León P, Miranda-Páez A, Chávez-Reyes J, Allende G, Barragán-Iglesias P, Marichal-Cancino BA (2021). The periaqueductal gray and its extended participation in drug addiction phenomena. Neurosci Bull.

[12] Mogoseanu D, Smith AD, Bolam JP (1994). Monosynaptic innervation of facial motoneurones by neurones of the parvicellular reticular formation. Exp Brain Res.

[13] Engmann AK, Bizzozzero F, Schneider MP, Pfyffer D, Imobersteg S, Schneider R (2020). The gigantocellular reticular nucleus plays a significant role in locomotor recovery after incomplete spinal cord injury. J Neurosci.

[14] Toor RUAS, Sun Q-J, Kumar NN, Le S, Hildreth CM, Phillips JK (2019). Neurons in the intermediate reticular nucleus coordinate postinspiratory activity, swallowing, and respiratory-sympathetic coupling in the rat. J Neurosci.

[15] Gao L, Liu S, Gou L, Hu Y, Liu Y, Deng L (2022). Single-neuron projectome of mouse prefrontal cortex. Nat Neurosci.

