# Supplementary material for: Preferential subcortical collateral projections of pedunculopontine nucleus-targeting cortical pyramidal neurons revealed by brain-wide single fiber tracing
Source: Mol Brain. 2022 Oct 29;15:88. doi: 10.1186/s13041-022-00975-y (PMC9618196; doi:10.1186/s13041-022-00975-y)
Supplement: Supplementary file 1 — Additional file 1: Materials and Methods, Supplementary figures S1, S2 and Table S1. Fig. S1. Microinjection of rAAV2-retro to the PPN of a CaMKIIα-Cre mouse. Fig. S2. Brain-wide distribution of PPN-projecting neurons. Table S1. Verification of preferred subcortical collateralization of PPN-projecting cortical areas based on mouse brain projectome data. [file 13041_2022_975_MOESM1_ESM.pdf]

Additional file 1

Preferential subcortical collateral projections of pedunculopontine nucleus-targeting cortical pyramidal neurons revealed by brain-wide single fiber tracing

Qiao-Qiong Liu, Yu-Xiao Cheng, Qi Jing, Ke-Ming Zhang, Lu-Feng Ding, Xiao-Wei Fan, Chun-Hui Jia, Fang Xu, Guo-Qiang Bi and Pak-Ming Lau

*Correspondence: plau@ustc.edu.cn or gqbi@ustc.edu.cn

This file includes:

Materials and Methods

Fig. S1

Fig. S2

Table. S1

List of additional files 2-8

List of abbreviations

Materials and Methods

Animals

Adult male and female mice (8-16 weeks) were used in the experiment. Tracing experiments were performed using the CaMKIIα-Cre line. All mice were group-housed with food and water accessible *ad libitum* under a 12-hour light/dark cycle (6:00-18:00). All procedures were performed according to the guidelines of the Animal Use and Care Committee of the University of Science and Technology of China.

Surgery and viral injections

The animals were deeply anesthetized by intraperitoneal injection of pentobarbital sodium solution (100 mg/kg). For the retrograde tracing experiment, a 40-50 nl cocktail of rAAV2-retro-EF1α-DIO-EGFP virus (BrainVTA; virus titer: 5.2×10^{12} vg/ml) and Alexa Fluor 647 conjugated-cholera toxin B subunit (Thermo Fisher Scientific, CTB-647, 0.02 mg/mL) was microinjected into the PPN with a glass micropipette at a rate of 5 nl/min under the control of a micropump (Nanoliter 2000 Injector, WPI) following the coordinates AP -4.27 mm, ML -1.27 mm, DV 2.95 mm [1,2]. For the sparse labeling experiment, diluted rAAV2-retro-EF1α-DIO-Flp (BrainVTA; virus titer: 1.6×10^{11} vg/ml) was microinjected into the PPN, and rAAV2/9-EF1α-fDIO-EYFP-EYFP (BrainVTA; virus titer: 5.0×10^{12} vg/ml) was microinjected into cortical regions (MOs: AP 2.46 mm, LM -0.5 mm, DV 0.9 mm; PL: AP 1.98 mm, LM -0.5 mm, DV 2.4 mm; ACA: AP 1.18 mm, LM -0.5 mm, DV 2.5 mm) [5,6].

Tissue processing

Mice were deeply anesthetized and sacrificed 3-4 weeks after surgery, followed by transcardial perfusion with 4% paraformaldehyde (PFA) in 0.1 M phosphate buffer. Brain tissues were isolated and immersed in 4% PFA overnight for post fixation. Then the brain tissues were embedded in an equal mixture of bovine serum albumin (BSA, 10% final concentration, Sigma) and hydrogel monomer solution (HMS) at 37°C for 4 hours. HMS contains 4% PFA (Electron Microscopy Sciences), 4% acrylamide (Sigma), 0.05% bisacrylamide (Sigma) and 0.0025% VA044 (Wako) in PBS (w/v).

Embedded tissues were sectioned into 43-47 pieces of 300-μm-thick slices with a vibroslicer (Compresstome VF-300, Precisionary Instruments) and cleared in 4% sodium dodecyl sulfate (SDS) for 4 hours at room temperature with gentle shaking.

Then, the slices were immersed in Hoechst solution (10 μg/ml) for 4 hours. Finally, the samples were rinsed in 0.1 M PBS three times before being mounted.

VISoR imaging and image processing

Brain slices were mounted on a quartz slide with polymerized HMS. Slices were immersed in a refractive-index-matching solution with a refractive index of 1.46 for 8 hours. Fluorescent images of the whole brain were acquired using the VISoR system at a resolution of $1 \times 1 \times 2.5\ \mu m^3$[3]. Image data were reconstructed automatically using custom software as previously described [4]. Adjacent slices were stitched together by nonrigid transformation followed by elastic deformation of local domains. Images were registered using custom software according to the Allen Mouse Brain Common Coordinate Framework (Allen CCF) [5]. In retrograde tracing experiments, cell counting was trained with ilastik software to identify and count the neurons in each 25 μm brain slice. These counting results were matched to the Allen CCF by custom software to obtain the number of neurons within each brain area. For fiber tracing, axons and dendrites were annotated semiautomatically using custom software implementing the Virtual Finger technology at 1-μm resolution. For validating the tracing pathway, each neuron was traced by two trained tracers and checked by the other more experienced annotator by merging the two tracing pathways. In total, we imaged 3 animals and traced the full morphology of 15 neurons, 11 of which were validated and used for further analyses.

Quantification and statistics

To assess whole-brain inputs to the PPN, the input proportion for each identified upstream brain area was calculated from the number of retrogradely labeled neurons in this area divided by the total number of labeled neurons in the whole brain. The mean and standard error of this value were calculated based on the data from 3 injected animals and were plotted in Fig. S2. For the single neuron collateralization analysis in Fig. 1i, the number of axonal termini (target number) in one brain area was used to quantify the density of the projection from each traced cortical neuron. For overall collateralization analysis of afferents received by different subcortical targets from the cortical areas (Fig. 1j), the collateralization ratio was defined as the number of traced neurons from a given cortical area, each having at least 2 termini in a specified subcortical target area divided by the total number of neurons traced for this cortical

area. The same definition was also used to quantify search results from the mouse brain projectome database [7]. From the database, the number of PPN-projecting neurons was obtained by searching for neurons with soma located in the MOs, ACA or PL, while having at least two termini in the PPN. The number of neurons that also project to a collateralization target identified in Fig 1i was obtained by adding this target area to the search criteria.

References

1. Tervo DGR, Hwang B-Y, Viswanathan S, Gaj T, Lavzin M, Ritola KD, et al. A Designer AAV Variant Permits Efficient Retrograde Access to Projection Neurons. Neuron. 2016; 92:372–82.

2. Zhu X, Lin K, Liu Q, Yue X, Mi H, Huang X, et al. Rabies Virus Pseudotyped with CVS-N2C Glycoprotein as a Powerful Tool for Retrograde Neuronal Network Tracing. Neurosci Bull. 2020; 36:202–16.

3. Wang H, Zhu Q, Ding L, Shen Y, Yang C-Y, Xu F, et al. Scalable volumetric imaging for ultrahigh-speed brain mapping at synaptic resolution. Natl Sci Rev. 2019; 6:982–92.

4. Shi M-Y, Ding L-F, Guo Y-H, Cheng Y-X, Bi G-Q, Lau P-M. Long-range GABAergic projections from the nucleus of the solitary tract. Mol Brain. 2021; 14:38.

5. Wang Q , Ding Sl, Li Y, Royall J, Feng D, Lesnar P, et al. The Allen Mouse Brain Common Coordinate Framework: A 3D Reference Atlas. Cell. 2020; 181:936-953.e.20.

6. Liu Q, Wu Y, Wang H, Jia F, Xu F. Viral Tools for Neural Circuit Tracing. Neurosci Bull. 2022; DIO:10.1007/s12264-022-00949-z.

7. Gao L, Liu S, Gou L, Hu Y, Liu Y, Deng L, et al. Single-neuron projectome of mouse prefrontal cortex. Nat Neurosci. 2022; 25:515-529.

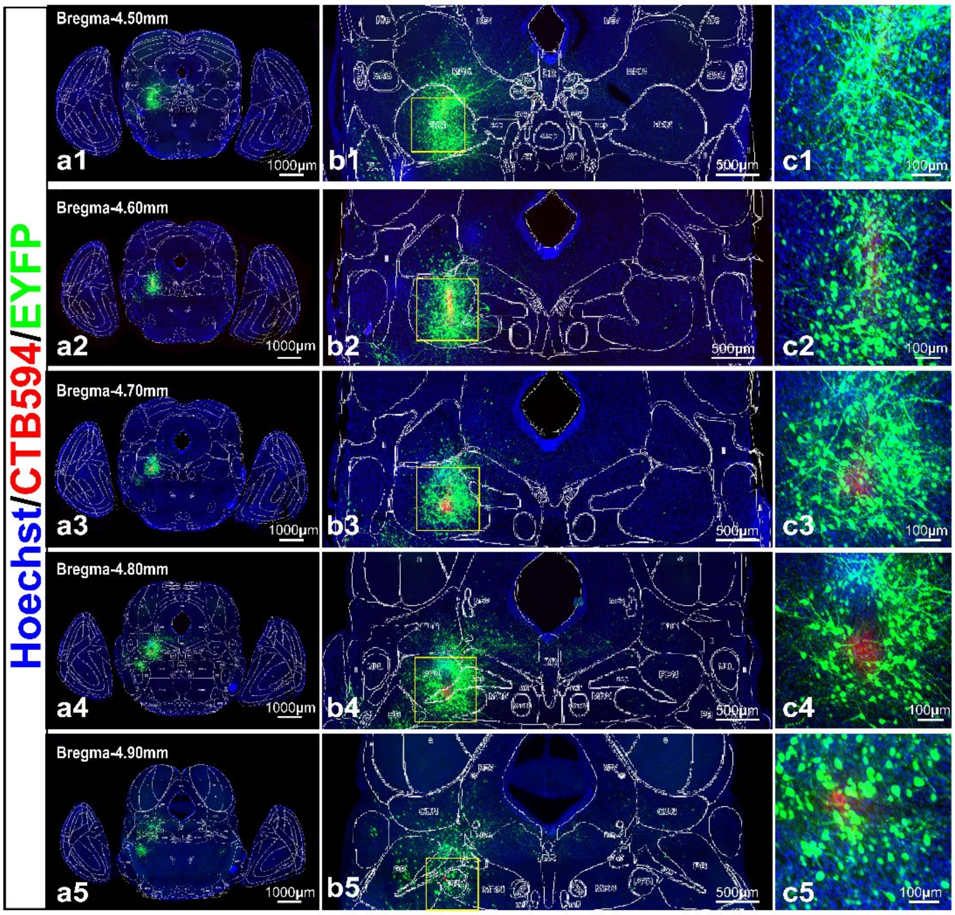

Fig. S1. Microinjection of rAAV2-retro to the PPN of a CaMKIIα-Cre mouse.

(a) Series of coronal brain sections of a CaMKIIα-Cre mouse injected with rAAV2-retro-EF1α-DIO-EGFP into the PPN, scale bar: 1000 μm. (b) Magnified views showing EGFP expression (green) with coinjected CTB-647 fluorescence (red) indicating the injection site that is limited within the PPN, scale bars: 500 μm; (c) Magnified views of boxed areas in b, scale bars: 100 μm.

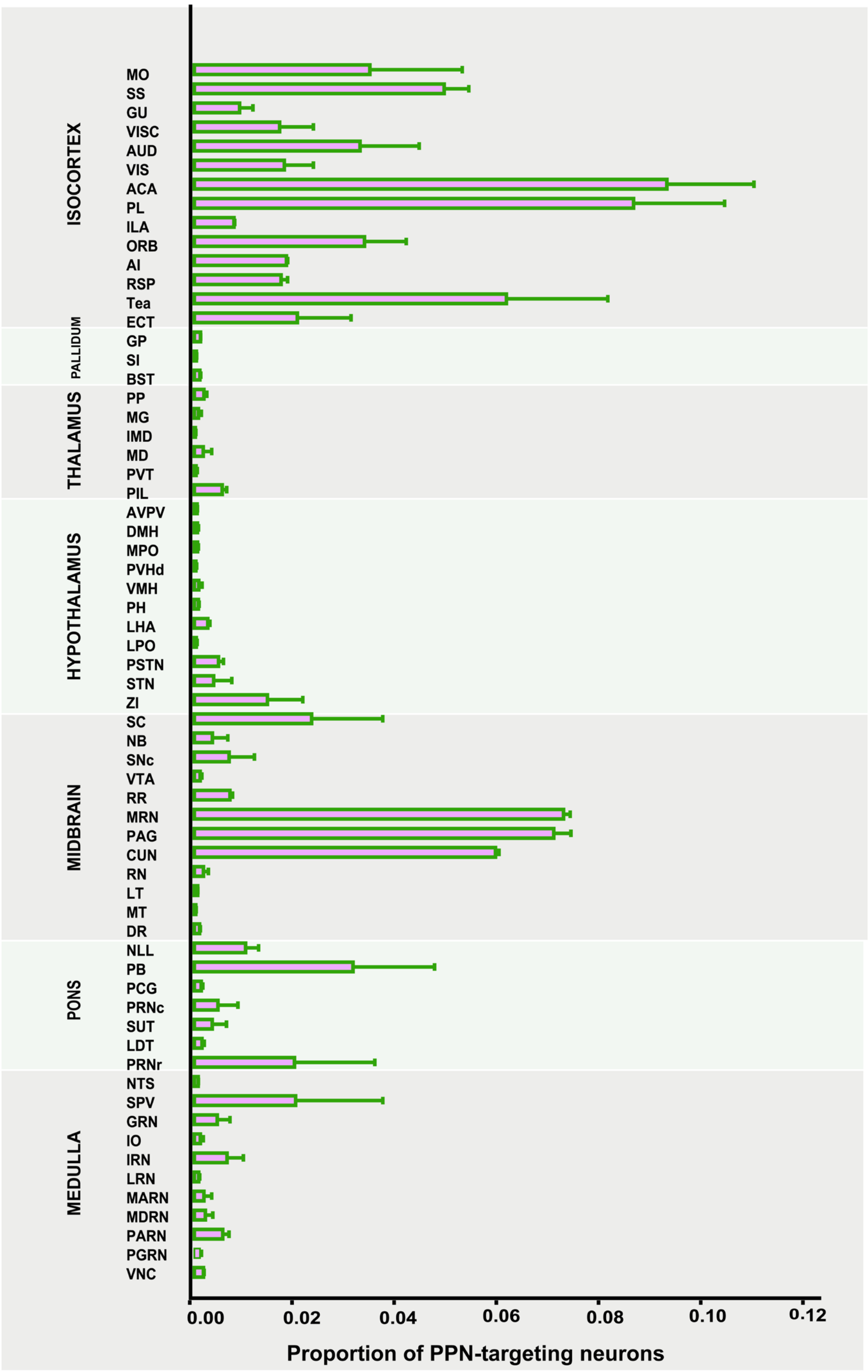

Fig. S2. Brain-wide distribution of PPN-projecting neurons. Three weeks after rAAV2-retro-EF1α-DIO-EGFP was injected into the PPN of CaMKIIα-Cre mice, the number of fluorescence labeled soma was counted in cortical and subcortical areas brain-wide (n=3).

Table S1 Verification of preferred subcortical collateralization of PPN-projecting cortical areas based on mouse brain projectome data

Collateral target	Collateralization ratio		
	ACA	PL	MOs
STR	0.99	0.96	0.96
LH	0.78	0.95	0.52
PAG	0.99	0.96	0.83
ZI	0.93	0.54	0.65
MRN	0.85	0.90	1.00
SNr	0.38	0.33	0.61
IRN	0.07	0.08	0.48
PARN	0.00	0.04	0.30
GRN	0.20	0.24	0.74

The collateralization ratio was calculated from the number of PPN-projecting neurons that also target the subcortical area (with at least 2 termini) divided by the total number of PPN-projecting neurons (with at least 2 termini each) from this cortical area in the database. The total number of PPN-projecting neurons: 101 from ACA, 167 from PL, 23 from MOs **[7]**.

Content of additional files 2-8

Additional file 2: Video S1. Whole-brain distribution of CaMKIIα afferent neurons to the PPN.

Additional file 3: Video S2. Axonal arborizations of ACA neurons in the PPN.

Additional file 4: Video S3. Axonal arborizations of ACA neurons in the PAG.

Additional file 5: Video S4. Axonal arborizations of MOs neurons in the PPN.

Additional file 6: Video S5. Axonal arborizations of MOs neurons in the striatum.

Additional file 7: Video S6. Axonal arborizations of PL neurons in the VTA.

Additional file 8: Video S7. Axonal arborizations of PL neurons in the PPN.

List of abbreviations

**MO**	Somatomotor areas
**MOs**	Secondary motor cortex
**SS**	Somatosensory areas
**GU**	Gustatory areas
**VISC**	Visceral area
**AUD**	Auditory areas
**VIS**	Visual areas
**ACA**	Anterior cingulate area
**PL**	Prelimbic area
**ILA**	Infralimbic area
**ORB**	Orbital area
**AI**	Agranular insular area
**RSP**	Retrosplenial area
**Tea**	Temporal association areas
**ECT**	Ectorhinal area
**STR**	Striatum
**GPI**	Globus pallidus, internal segment
**GP**	Globus pallidus
**TU**	Olfactory tubercle
**SI**	Substantia innominata
**BST**	Bed nuclei of the stria terminalis
**PP**	Peripeduncular nucleus
**MG**	Medial geniculate complex
**IMD**	Intermediodorsal nucleus of the thalamus
**VM**	Ventromedial thalamic nucleus
**VPM**	Ventral posteromedial thalamic nucleus
**PF**	Parafascicular nucleus
**MD**	Mediodorsal nucleus of thalamus
**PVT**	Paraventricular nucleus of the thalamus
**PIL**	Posterior intralaminar thalamic nucleus
**AVPV**	Anteroventral periventricular nucleus
**DMH**	Dorsomedial nucleus of the hypothalamus
**MPO**	Medial preoptic area
**PVHd**	Paraventricular hypothalamic nucleus, descending division
**VMH**	Ventromedial hypothalamic nucleus
**PH**	Posterior hypothalamic nucleus
**LHA**	Lateral hypothalamic area
**LPO**	Lateral preoptic area
**PSTN**	Parasubthalamic nucleus
**STN**	Subthalamic nucleus
**ZI**	Zona incerta
**SC**	Superior colliculus
**SCm**	Superior colliculus, motor related
**NB**	Nucleus of the brachium of the inferior colliculus
**SNr**	Substantia nigra, retiular part
**SNc**	Substantia nigra, compact part
**VTA**	Ventral tegmental area
**FF**	Fields of Forel

**PPN**	Pedunculopontine tegmental nucleus
**RR**	Midbrainreticular nucleus, retrorubral area
**MRN**	Midbrain reticular nucleus
**PAG**	Periaqueductal gray
**CUN**	Cuneiform nucleus
**RN**	Red nucleus
**MBO**	Mammillary body
**MA3**	Medial accessory oculomotor nucleus
**NPC**	Nucleus of the posterior commissure
**LT**	Lateral terminal nucleus of the accessory optic tract
**MT**	Medial terminal nucleus of the accessory optic tract
**DR**	Dorsal nucleus raphe
**NLL**	Nucleus of the lateral lemniscus
**PB**	Parabrachial nucleus
**PG**	Pontine gray
**P5**	Peritrigeminal zone
**PB**	Parabrachial nucleus
**CS**	Superior central nucleus raphe
**TRN**	Tegmental reticular nucleus
**LC**	Locus ceruleus
**PCG**	Pontine central gray
**PRNc**	Pontine reticular nucleus, caudal part
**SUT**	Supratrigeminal nucleus
**LDT**	laterodorsal tegmental nucleus
**PRNr**	Pontine reticular nucleus
**SPV**	Spinal nucleus of the trigeminal
**GRN**	Gigantocellular reticular nucleus
**IO**	Inferior olivary complex
**IRN**	Intermediate reticular nucleus
**LRN**	Lateral reticular nucleus
**MARN**	Magnocellular reticular nucleus
**MDRN**	Medullary reticular nucleus
**PARN**	Parvicellular reticular nucleus
**NTS**	Nucleus of the solitary tract
**PGRN**	Paragigantocellular reticular nucleus
**PMR**	Paramedian reticular nucleus
**VNC**	Vestibular nuclei
**MV**	Medial vestibular nucleus
